# A Comparison Between Body Weight-Supported Treadmill Training and Conventional Over-Ground Training in Dogs With Incomplete Spinal Cord Injury

**DOI:** 10.3389/fvets.2021.597949

**Published:** 2021-07-01

**Authors:** Ângela Martins, Débora Gouveia, Ana Cardoso, Inês Viegas, Óscar Gamboa, António Ferreira

**Affiliations:** ^1^Arrábida Veterinary Hospital—Animal Rehabilitation Center, Azeitão, Portugal; ^2^Faculty of Veterinary Medicine, Lusófona University, Lisboa, Portugal; ^3^CIISA—Centro Interdisciplinar de Investigação em Saúde Animal—Faculty of Veterinary Medicine, Lisboa, Portugal; ^4^Faculty of Veterinary Medicine, University of Lisbon, Lisboa, Portugal

**Keywords:** dogs, rehabilitation, treadmill, over-ground training, spinal cord injury

## Abstract

In human medicine there was no evidence registered of a significant difference in recovery between body weight-supported treadmill training (BWSTT) and conventional over-ground (COGI). There isn't any similar study in veterinary medicine. Thus, this study aimed to compare the locomotor recovery obtained in incomplete SCI (T11–L3 Hansen type I) post-surgical dogs following BWSTT or COGI protocols, describing their evolution during 7 weeks in regard to OFS classifications. At admission, dogs were blindly randomized in two groups but all were subjected to the same protocol (underwater treadmill training) for the first 2 weeks. After, they were divided in the BWSTT group (*n* = 10) and the COGI group (*n* = 10) for the next 2 weeks, where they performed different training. In both groups locomotor training was accompanied by functional electrical stimulation (FES) protocols. Results reported statistically significant differences between all OFS evaluations time-points (*p* < 0.001) and between the two groups (*p* < 0.001). In particular with focus on T1 to T3 a two-way repeated measures ANOVA was performed and similar results were obtained (*p* = 0.007). Functional recovery was achieved in 90% (17/19) of all dogs and 100% recovered bladder function. The BWSTT group showed 100% (10/10) recovery within a mean time of 4.6 weeks, while the COGI group had 78% (7/9) within 6.1 weeks. Therefore, BWSTT leads to a faster recovery with a better outcome in general.

## Introduction

Intervertebral degenerative hernia (IVDH) is the most common cause of acute spinal cord injury (SCI) in dogs, with a prevalence as high as 19% in some breeds (1–3). The most prevalent type of IVDH is extrusion thoracolumbar IVDH Hansen type I (4). IVDH can occur in any breed of dogs (5), but it typically affects chondrodystrophic dogs at an age ranging from 3 to 6 years. However, it also occurs in non-chondrodystrophic dogs (6, 7).

The clinical signs of IVDH vary, ranging from spinal hyperesthesia to hindlimb paralysis (8, 9). Severe clinical signs that imply the loss of voluntary motor function after acute SCI are mostly caused by contusion (7). IVDH Hansen type I results in mixed contusive and compressive injury (10–12) and causes both primary and secondary lesions (13). This condition can benefit from decompressive surgery (14), particularly in the case of dogs with rapidly deteriorating neurological status (15).

Moore et al. reported that 43% of veterinarians routinely recommend a postsurgical rehabilitation program, with the treatment beginning immediately after surgery. It has been reported that nursing care of these dogs benefits their recovery of ambulation. In addition, there are many diverse recommendations, ranging from no restriction to strict cage rest up to 8 weeks post-surgery (3).

In human medicine, rehabilitation strategies aim to improve stepping recovery after SCI (16). Locomotor training exercises with step repetitions for several minutes each day for several consecutive weeks improve muscle force output and endurance (17) and also promote functional plasticity in locomotor central pattern generators (CPGs) present in the spinal network (18).

Locomotor training based on repetitions also affects the characterization and quality of gait through more complex mechanisms, such as the stimulation of residual descending/ascending pathways. Thus, locomotor training facilitates the recovery of ambulation after incomplete SCI (17).

The spinal cord depends on sensory feedback to generate force during stepping. In cats with spinal injury, a direct access between the cutaneous inputs and spinal rhythm-generating circuitry is suggested and treadmill training can provide 50% of the force generated during the stance phase (19).

In humans, high-intensity locomotor training promotes the release of various trophic factors, such as brain-derived neurotrophic factor (BDNF), which may facilitate angiogenesis, neurogenesis, neuro-reorganization changes, and muscle metabolism (20, 21). This type of training in humans with SCI includes body weight-supported treadmill training (BWSTT), which plays an important role in posture and gait (22). In addition, BWSTT can increase the amplitude and coordination of firing of muscle motor units (23).

Dobkin et al., compared gait evolution by BWSTT and by conventional over-ground intervention (COGI) and found that there was no significant difference between the two treatments up to 3 months (24). Thus, BWSTT did not differ significantly from COGI in terms of kinematic and kinetic parameters (25–27). A study by Field-Fote and Roach indicated that gains in walking speed obtained with COGI were equivalent to gains achieved with BWSTT and that regarding walking distance, COGI was superior (28).

An association between functional electrostimulation (FES) and locomotor training that improves normality in speed, distance, and limb coordination has been reported (29). In addition, FES improves stride, length, cadence, and gait quality, as well as muscle strength and activation (29, 30). FES with locomotor training is implemented in individuals with complete or incomplete SCI (31). This electrostimulation modality, targets restorative neurology by modifying residuals and remaining nervous system circuits and pathways (32).

Given that rehabilitation protocols are already successfully used in humans, it is considered to be a therapy with reasonable translational potential for veterinary medicine (33). Thus, this study aimed to compare the application of the BWSTT protocol vs. the COGI protocol in dogs with incomplete sub-acute SCI (T11–L3 IVDH Hansen type I) after surgery.

## Materials and Methods

### Participants

This was a controlled, blinded, prospective clinical study using a cohort criterion in dogs. Dogs presenting with an acute history of thoracolumbar SCI with IVDH Hansen type I after hemilaminectomy at one or two sites were included. The time between onset of disease and surgery was ~2–4 days, during which they were treated with a pharmacological approach with non-steroidal anti-inflammatories or with low doses of corticosteroids. All dogs were diagnosed using computerized tomography (CT) or magnetic resonance imaging (MRI) with T11–L3 neuro-localization.

All modified Frankel Scale (MFS) grade I dogs, regardless of sex and breed, were included in this study and were admitted to the rehabilitation center up to 7 days postoperatively. Other inclusion criteria were weight ≤ 20 kg and age from 2 to 8 years.

At admission, all participants underwent a neurorehabilitation functional examination and a standard upper motor neuron examination and showed decreased or absent withdrawal reflexes in one or both pelvic limbs. This status, in the absence of an SCI in the lumbosacral intumescence, was compatible with spinal shock diagnosis, which can be associated with disk herniation (34).

All subjects demonstrated a neurogenic bladder observed at admission, which was compatible with SCI neuro-localization: cranial to the spinal cord segment S1 or cranial to the L4 vertebra, resulting in increased urethral resistance and difficulties in vesical expression (35).

This study was performed between November 2017 and January 2020 at the Arrábida Veterinary Hospital Rehabilitation Center after approval by the Lisbon Veterinary Medicine Faculty ethics committee.

Dogs were excluded from this study if they had suspected or confirmed spinal cord neoplasia, myelomalacia phenomenon, meningomyelitis, IVDH Hansen type II, thoracolumbar fibrocartilaginous embolic myelopathy, acute non-compressive nucleus pulposus extrusion, or thoracolumbar myelitis. They were also excluded if they were diagnosed with IVDH Hansen type I but had different neuro-localization and/or had normal/increased withdrawal reflexes. Dogs with T11–L3 IVDH Hansen type I were excluded if they underwent conservative management and/or if they were admitted to the center more than 7 days postoperatively.

### Study Design

Twenty-two subjects were admitted to the study and underwent a neurorehabilitation examination according to clinical history and physical and neurologic examination after the owners provided consent.

All subjects had an alert mental status, passive standing posture, diverse postures (kyphosis and scoliosis), upper motor neuron peripheral reflexes with the exception of the withdrawal reflex (absent/decreased in one or both limbs), and cutaneous trunci reflexes present at or below the injury site. Overall, superficial and deep pain perception was present.

Deep pain perception was evaluated in a controlled environment, with respect to movement and noise, with 12-cm Halsted mosquito forceps, in both medial/lateral digits of both pelvic limbs. For a more accurate pain perception examination, this was also tested in the perineal region and at the tip and base of the tail.

Spinal hyperesthesia was also assessed with the correct technique of vertebral column palpation. This technique is performed by placing slight pressure on the vertebral column, placing one hand on the abdominal muscles, palpating throughout the dorsal longitudinal ligament between each spinous process, and between each intervertebral space, in order to activate the nociceptors located in the peripheral fibrous annulus (36).

With the dogs in a standing posture, both pelvic limbs were palpated to compare symmetry and tonus. All dogs showed hypotonicity in the flexor muscle group. Proprioceptive positioning was evaluated in dogs while supported by the examiner.

This study used the Open Field Score (OFS) (37) to quantify recovery of stepping ability and to observe coordination between the thoracic and pelvic limbs.

Videotaping was performed on admission (T0) and 2 weeks later (T1). Dogs were randomized into two groups. Dogs were randomly assigned to groups according to the order of admission in the center, and the first selected dog was designated by a blinded procedure.

Evaluations were performed and recorded each week from T1 (T2 to T6) and at follow-ups (F1, F2, and F3). Videotapes were analyzed and scored by two blinded observers, board-certified canine rehabilitation veterinarians, and if interobserver disagreement exceeded 20%, a third blinded observer, working at a different institution, performed the same analysis.

Twenty-two dogs were admitted in the study and were subjected to the same protocol of functional neuro-rehabilitation (FNR) during the first 2 weeks. The protocol of FNR included: Underwater treadmill (FNR-UWTM); IES and FES.

After the first 2 weeks, they were allocated to different groups, but two dogs left the study before randomization due to the owners' decision. The remaining twenty dogs (*n* = 20) were separated into the FNR-BWSTT protocol group (*n* = 10) and the FNR-COGI protocol group (*n* = 10). Both groups performed these protocols during the next 2 weeks and had outcomes observed at T2 and T3, based on neurological examination and OFS inter-observer observations.

**Figure 1:**
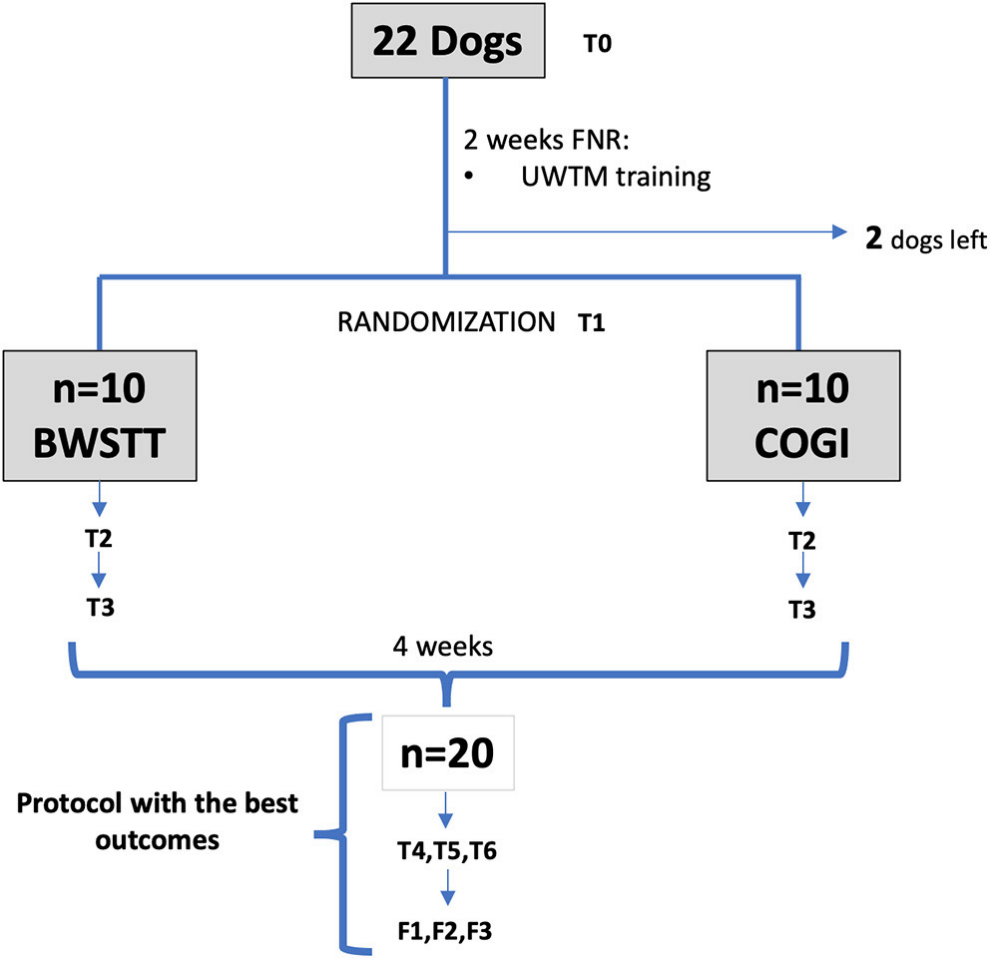
Flow diagram describing the study design. FNR, Functional neurorehabilitation; UWTM, Underwater treadmill; T0, Admission; T1, After 2 weeks, randomization time; T2, After 1 week of specific protocol; T3, After 2 weeks of specific protocol; T4, T5, T6, After 3,4, and 5 weeks of the same protocol; F1, F2, F3, Follow-up after 1 week, 1 and 2 months. BWSTT, Body weight-supported treadmill training; COGI, conventional over-ground intervention.

The group with the lower success rate after these first 4 weeks of FNR, was transferred to the protocol with higher results, and evaluations were performed at T4, T5, and T6. As can be seen in Figure 1 the diagram of this study within a 7-week period is described and explains the 2-week interval between T0 and T1, and also the evaluations at T2 (3rd week), T3 (4th week), T4 (5th week), T5 (6th week), and T6 (7th week).

### Interventions

The 22 dogs that were included in the study started the FNR-UWTM protocol within 24 h after admission. The 1st day was considered an adaptation to all neurorehabilitation interventions.

#### FNR-UWTM Protocol

In the first 2 weeks (T0-T1), all dogs received interferential electrical stimulation (IES) for pain management throughout a 4-channel electrotherapy device (BTL-5645 PULS, BTL, USA). IES is a pre-modulated interferential current commonly used in canine rehabilitation.

**Figure 2:**
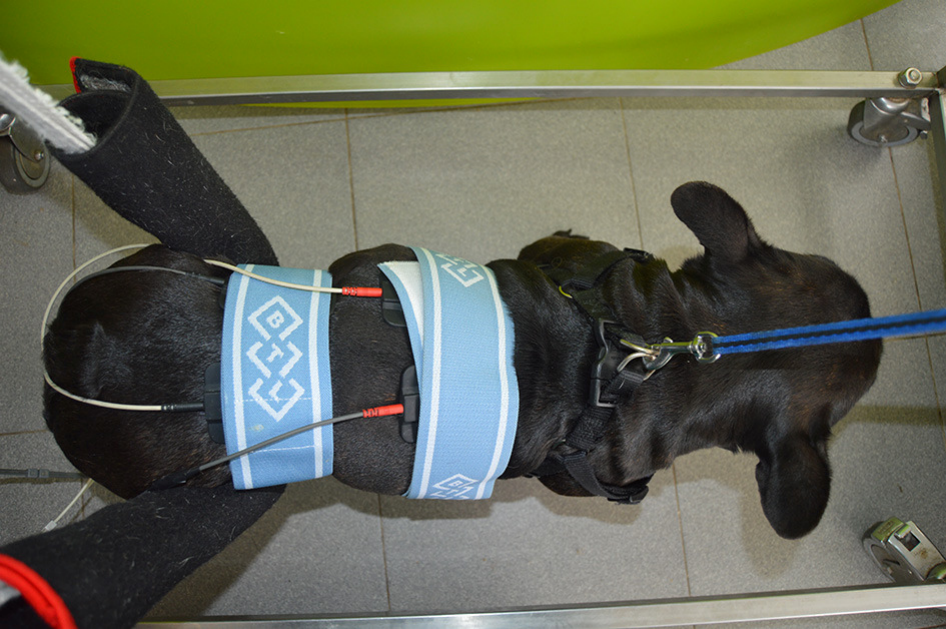
Interferential electrical stimulation (IES) for pain management in postural standing position. Application of two different channels with four electrodes in a crossed position.

This technique used two different channels with four rubber and carbon electrodes (7 × 5 cm) (BTL, USA) placed crossing each other at a 90° angle and located proximately two spaces cranial and caudal to the spinal hyperesthesia site, after clipping the hair and application of gel (Figure 2). The current applied was biphasic, symmetric and continuous and was performed two times/day, 5 days/week in the 1st week. On the 2nd week this was applied once a day, each 48 h. Pain level was assessed daily by a correct technique of vertebral column palpation and recorded to better evaluation in slow motion mode.

Current parameters were applied as followed:

- Channel 1 (Acute pain)—Frequency was 80–150 Hz; Intensity was 0.5–1 mA; Pulse duration was 2–50 μs; Time of treatment of 10 min (38, 39);
- Channel 2 (Chronic pain)—Frequency was 1–10 Hz; Intensity was 0.5–1 mA; Pulse duration was 100–400 μs; Time of treatment of 10 min (38, 39).

**Figure 3:**
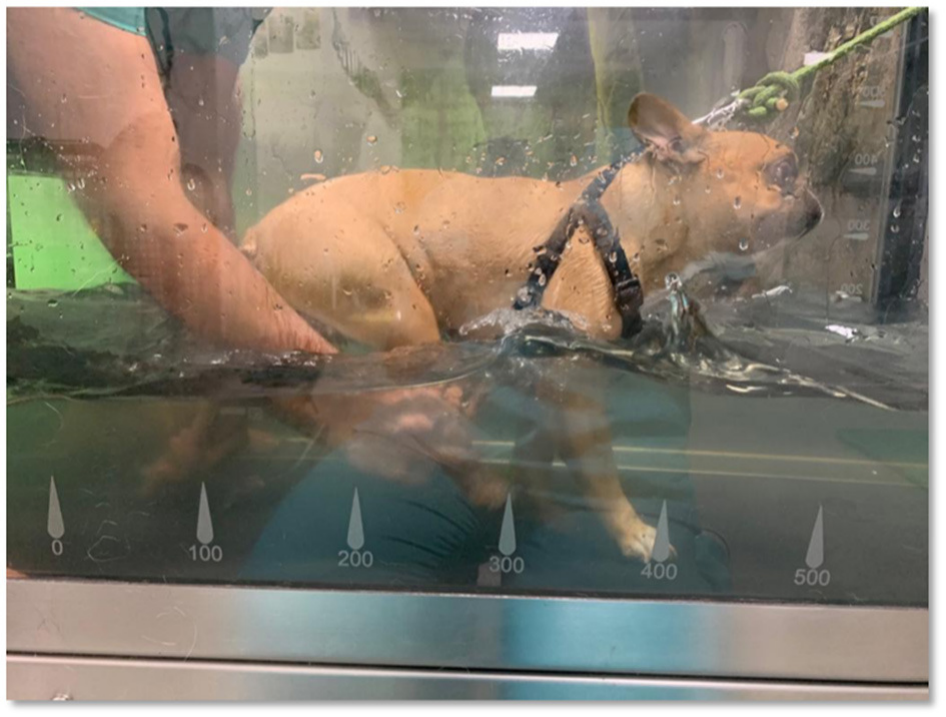
Underwater treadmill locomotor training, with pelvic limb bicycle movements performed by the technician.

Participants were also subjected to locomotor training based on repetitive water training, with established pelvic limb bicycle movements, stretching of pelvic limb muscles, and stimulation of cutaneous receptors on the treadmill surface (Figure 3). This exercise was alternated with perineal stimulation to obtain the flexion and extension cycles of small amplitude used for paralysis of the hindlimbs (40). All exercises were performed with weight support according to the patient's capacity at the time.

In the 1st days of FNR-UWTM training, each patient was allowed to adapt, with speeds beginning at ≤ 0.8 km/h and progressively increasing to 1.2, 1.9, and 2.5 km/h (41, 42), first for 3–10 min and extending to 20 min once a day within the first 2 weeks.

Water temperature was maintained between 24 and 26°C, and the water level was positioned between the lateral malleolus of the tibia and the lateral condyle of the femur (43). Dogs were monitored by assessing the mucous membranes, capillary refill time, and pulse.

The use of combined approaches in rehabilitation may be more effective than a single intervention, simultaneously targeting different injury mechanisms (44). Therefore, all participants underwent FES within this period with a 2-channel electrotherapy device (BTL-5645 PULS, BTL, USA).

FES is described to use low pulse frequency electrical currents to restore motor functions after SCI, promoting control standing, balance, posture and gait training. Also, increase tonus in the hamstring muscle and polysynaptic reflex (45), essential in this population of dogs that showed decreased or absent withdrawal reflexes.

**Figure 4:**
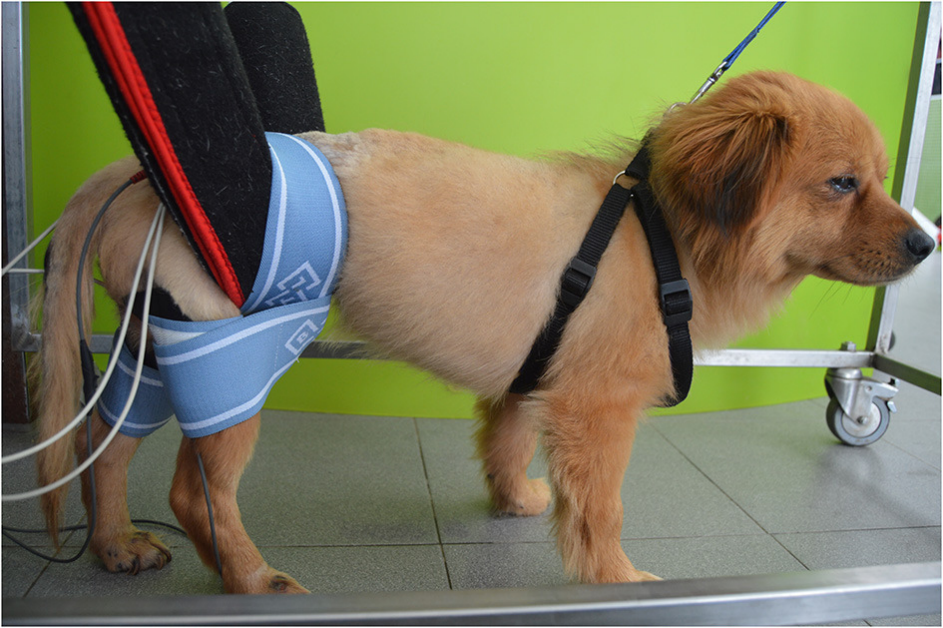
Functional electrical stimulation (FES) through the sciatic nerve. Superficial electrodes applied in a segmental technique, placed on the sciatic nerve root (L7-S1 region) and near the flexor muscle motor point.

Superficial rubber and carbon electrodes (7 × 5 cm) (BTL, USA) were applied after trichotomy in a segmental technique and were placed over the peripheral nerve trunk and motor point (46) with patients in the standing position (Figure 4). Thus, the main hamstring muscles were stimulated (biceps femoris, semitendinosus and semimembranosus), both cranial and caudal groups, allowing the stimulation of the stance and swing phase, respectively.

This was a symmetric, biphasic and pulsed alternating current with the following parameters: Frequency of 30 Hz (47); intensity of 6–16 mA (48); pulse duration 100–150 μs (49); duty cycle 1:4 ratio, 2–4 s ramp-up, 8 s on-time, 1–2 s ramp-down (38), and time of treatment of 10 min. FES was performed 2–3 times/day for 5 days/week.

During treatment, parameters were adjusted according to the monitoring of muscle fatigue signs, such as muscle weakness, fasciculations, increased respiratory rate and/or pain. In case any of these signs appeared, parameters were adjusted: intensity (mA) was reduced until they disappear and if they continued, frequency (Hz) was also reduced. Also, if they persist in time, duty cycle was increased (i.e., until 1:6).

#### FNR-BWSTT Protocol

In human medicine, BWSTT can be manually or robotically assisted, such as via the Lokomat system, which supports 60% of an individual's weight at the beginning, decreasing in accordance with load tolerance to no <25% support (50). A similar manual support approach was implemented in our dogs.

**Figure 5:**
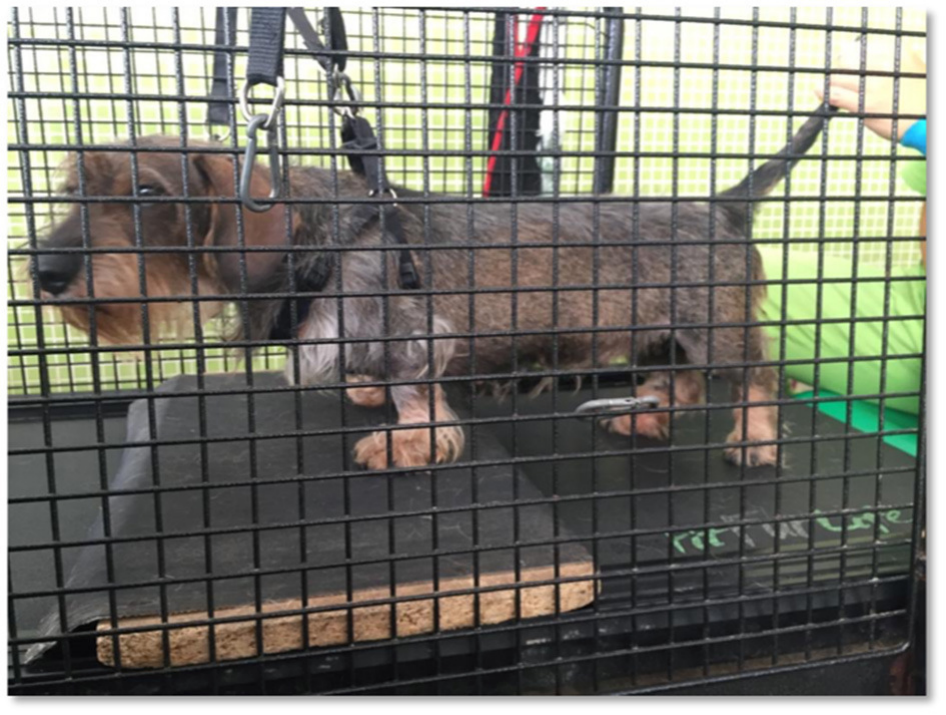
Body weight-supported treadmill training with bipedal step training. Forelimbs stationary on a fixed platform above the belt.

As all dogs were acclimated to the underwater treadmill, very few dogs resisted treadmill training. However, in dogs that offered resistance, bipedal step training was required, wherein the forelimbs remained stationary on a fixed platform above the belt (51) (Figure 5).

**Figure 6:**
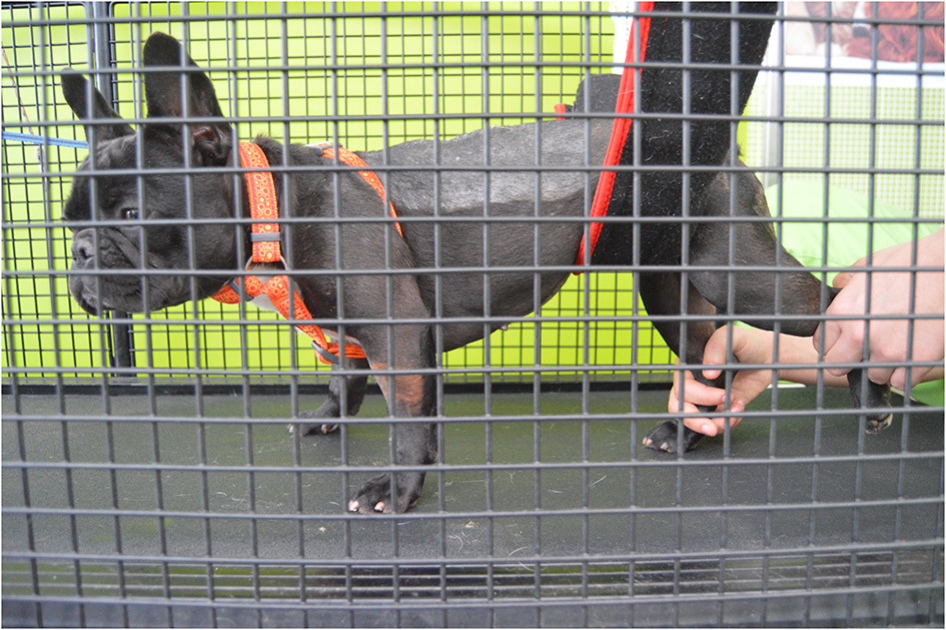
Body weight -supported treadmill training with quadrupedal step training. Technician preforming bicycle movements.

The aim of the BWSTT protocol was to achieve quadrupedal step training, starting with:

- Speeds of 1–2.5 km/h, during 20–30 min, 2–3 times/day, 6 days/week according to patients' capabilities (Figure 6) (52–54);
- The treadmill slope was elevated from 10 to 25°, with thoracic and pelvic limb coordination (55, 56).

All 10 dogs in this group underwent BWSTT within a therapeutic window of 40 min after application of FES, using the same technique as described earlier.

During these 2 weeks (T1-T3) it was performed the FNR-BWSTT protocol without alteration regarding time, frequency during the day and frequency during the week. Thus, the locomotor training had a total range from 40 to 90 min plus 20–30 min of FES in a daily basis.

#### FNR-COGI Protocol

At the same time (T1-T3), COGI was applied in 10 dogs, by one or two veterinarians' technicians, with the help of a harness system that allowed the dogs to perform step movements with similar weight support as described before, decreasing the load tolerance according to dogs' abilities.

During gait stimulation, all dogs had to touch the floor surface, beginning with a knuckling position and progressing to cutaneous receptor stimulation (placed in the interdigital space).

**Figure 7:**
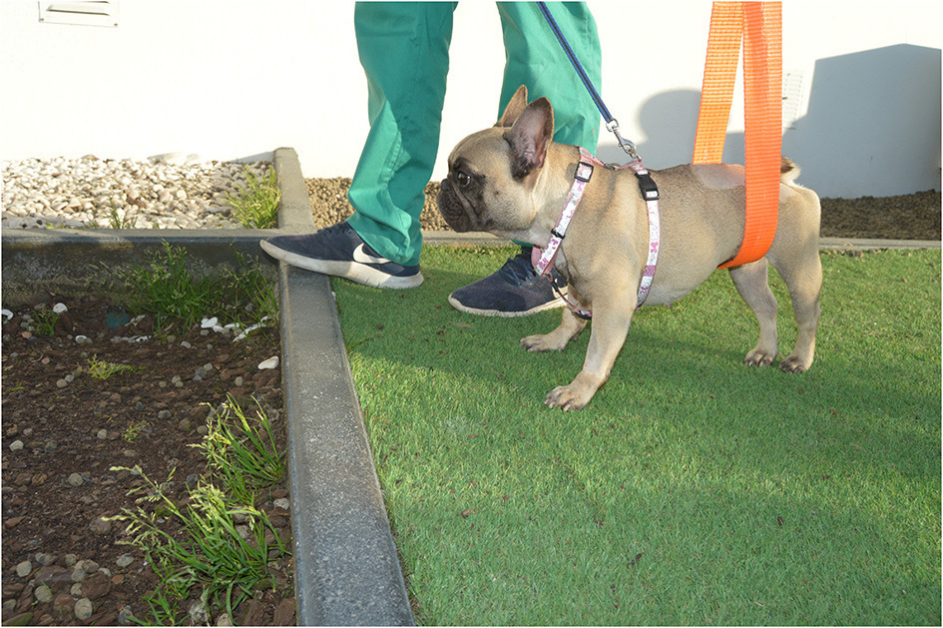
Conventional over ground intervention. Gait Stimulation in different floors surfaces, such as sand, stones, and grass.

Dogs were stimulated to walk for 20–30 min, 2–3 times/day, 6 days/week on different floor surfaces, such as sand, stones, and grass (Figure 7).

**Figure 8:**
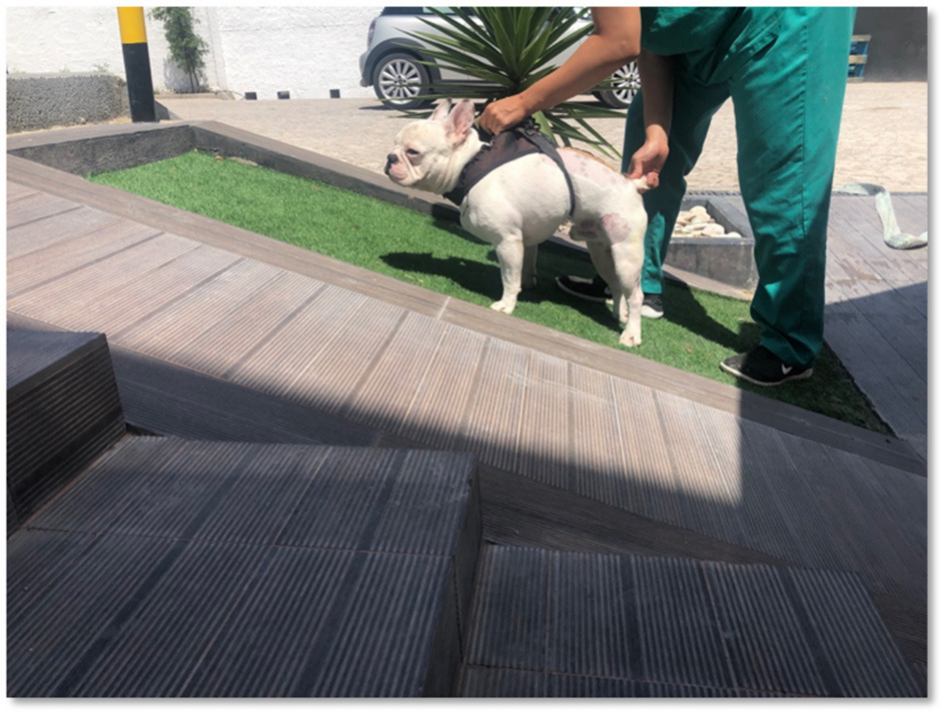
Gait Stimulation on uneven floor, such as ramp up and down.

After the dogs achieved a flexion–extension locomotor pattern, it was associated with gait stimulation on an uneven floor as well as up and down ramps (Figure 8), for the same amount of time.

All 10 dogs were subjected to the same FES protocol (Table 1) before they were stimulated to walk. Dogs performed a daily locomotor training from 40 to 90 min total and 20–30 min of FES during these 2 weeks.

**Table 1:** Functional Electrical Stimulation (FES) protocol.

Current	Symmetric
	Biphasic
	Pulsed
Frequency	30 Hz
Intensity	6–16 mA
Pulse duration	100–150 μs
Duty cycle	1:4
Shape	2–4 s ramp-up, 8 s on-time, 1–2 s ramp-down
Time of treatment	10 min; 2–3 times/day for 5 days/week

Thus, in both protocols (COGI and BWSTT), exactly the same time of CNS stimulation was promoted.

#### Supportive Care

Dogs were hospitalized full-time, resting in soft beds to avoid pressure sores or decubital ulcers, and doughnut bandages were placed under bone prominences. If they were recumbent, they were turned every 4–6 h from left to right lateral recumbency to prevent atelectasis or accumulation of lung secretions (57). They were always encouraged to maintain sternal recumbency.

Dogs were fed three times per day with an intake of 30% and hydric support of 100–120 ml/kg. All had neurogenic bladders, which were expressed every 4–6 h as a daily rule (57, 58).

#### Outcomes/Follow-Ups

All dogs underwent an FNR examination and an OFS evaluation every 7 days. The first evaluation was performed at admission (T0), followed by an evaluation at 2 weeks after initiating FNR-UWTM (T1). At this stage, the presence of the withdrawal reflex and crossed extensor reflex was evaluated, as the main outcome.

Blinded randomization was implemented at T1, when the dogs were divided into two groups: the BWSTT group and the COGI group. Evaluations were performed weekly over the subsequent 5 weeks (T2–T6), finishing the study within a 7-week period.

If neurological evolution was not evident in one of these groups until the finish of T3, the protocol was switched to the one that yielded the best apparent results. Thus, from T4 to T6, all dogs were treated with the same FNR protocol.

Follow-up was advised after the 1st week of release, also after 1 and 2 months. All were evaluated (FNR exam and OFS score) and videotaped.

In this study, functional outcome was considered when dogs achieved an OFS score classification of ≥ 11. An OFS of 11 is equivalent to a locomotor state in ambulatory paraparesis and OFS > 11 is equivalent to that in proprioceptive ataxia. A functional state can be defined as the possibility of a dog standing up, maintaining an active standing posture, giving five or more steps, and having the ability to urinate and defecate voluntarily.

### Statistical Analysis

Quantitative, qualitative, and categorical data were analyzed using IBM SPSS Statistics software, Version 22 (International Business Machines Corporation, Armonk, NY, USA), and the results were interpreted at a level of significance of *p* < 0.05.

For inter-observer validation regarding OFS scores, normality tests were performed, and normality was not verified. Thus, independent-samples Mann–Whitney *U*-tests were performed.

For comparison between groups and between evaluation time-points, a univariate analysis of variance was performed. The statistical model was considered significant (*p* < 0.001). Estimated marginal means with 95% confidence interval and an interaction plot for the comparison of mean OFS at each time-point between groups were derived.

Also, for better evaluation between T1 and T3, a two-way repeated measures ANOVA with time as a repeated measure, was performed. Thus, a measured dependent variable (OFS) was evaluated regarding two independent variables (time points and different protocols), with the primary purpose of understanding if there is an interaction between these two factors on the dependent variable.

## Results

**Figure 9:**
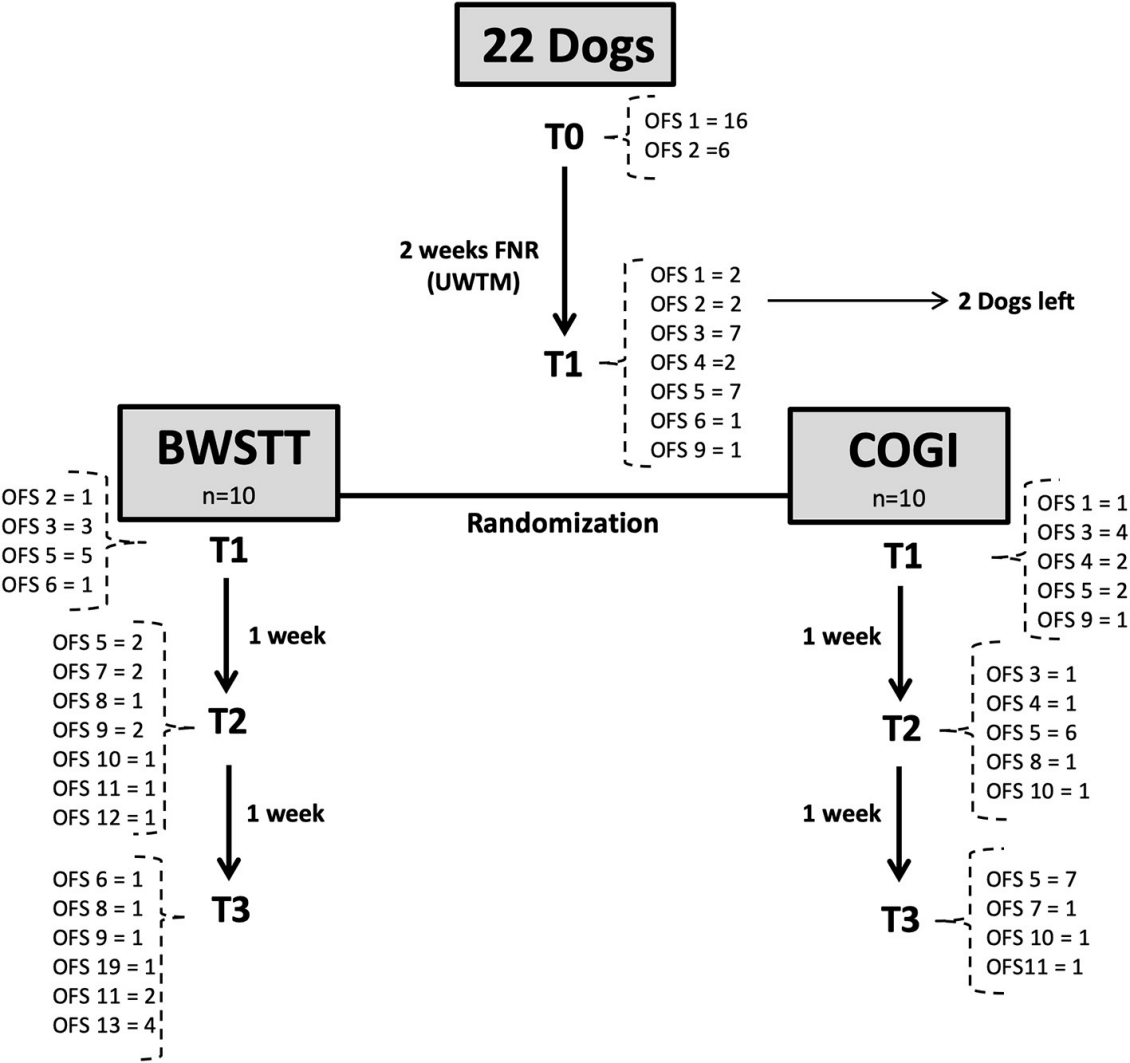
Flow diagram describing the neurologic evolution of the 22 dogs. FNR, Functional neurorehabilitation; UWTM, Underwater treadmill; T0, Admission; T1, After 2 weeks, randomization time; T2: After 1 week of specific protocol; T3, After 2 weeks of specific protocol; BWSTT, Body-weight supported treadmill training; COGI, conventional overground intervention.

**Figure 10:**
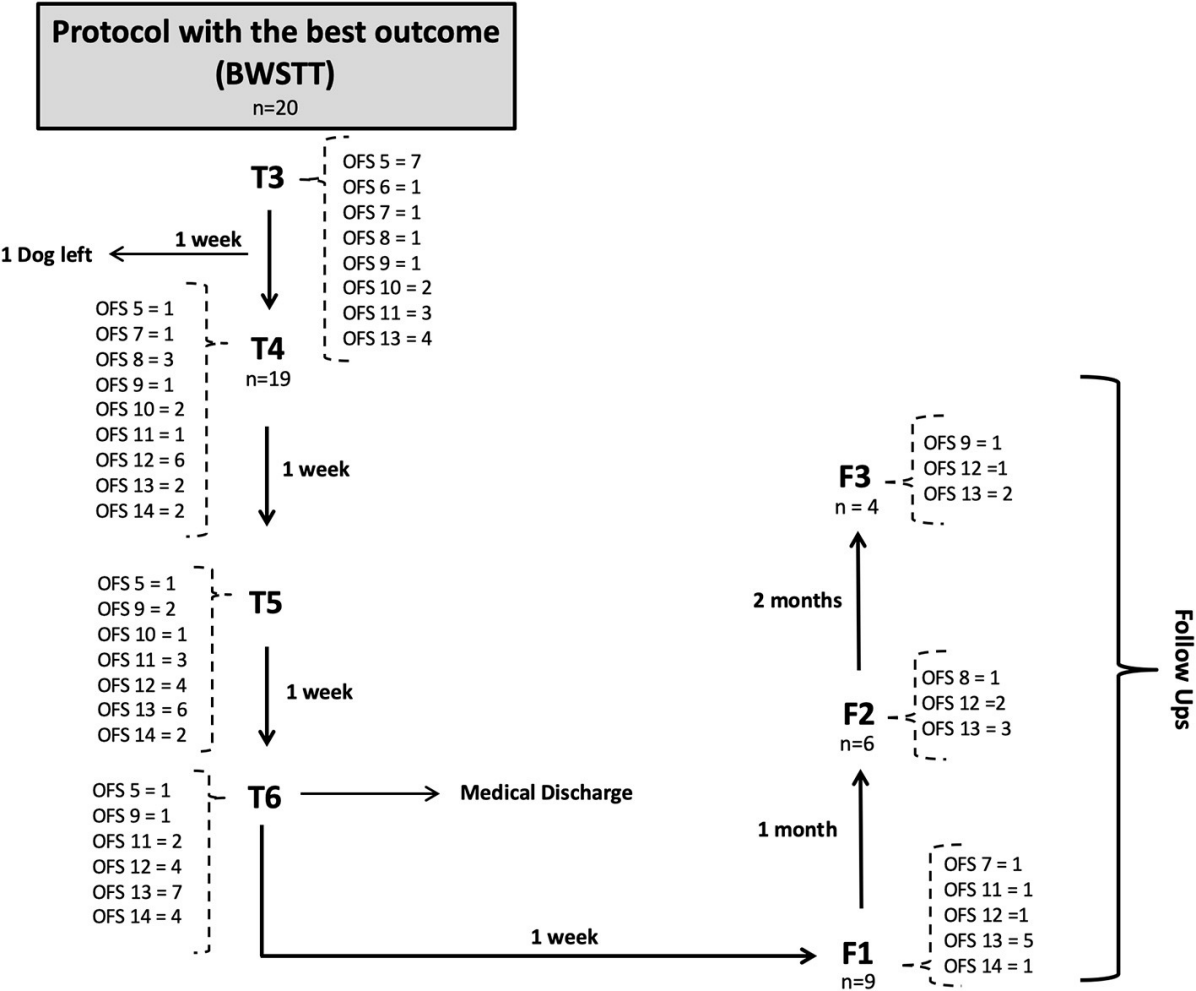
Flow diagram describing the neurologic evolution of the 22 dogs. FNR, Functional neurorehabilitation; T4, T5, T6,After 3,4 and 5 weeks of the same protocol; F1, F2, F3, Follow-up after 1 week, 1 and 2 months; BWSTT, Body-weight supported treadmill training; COGI, conventional overground intervention.

This study described the evolution of progress in 22 dogs throughout their FNR protocol over a 7-week period, according to the diagram in Figures 9, 10, and their evaluation using the OFS score.

In the total population (*n* = 22), the average age was 5.3 years and average weight was 9.65. There were 10 males and 12 females; 63.6% (14/22) were chondrodystrophic dogs and 36.4% (8/22) of non-chondrodystrophic dogs. The following was the breed distribution: six Dachshunds, four French Bulldogs, four Mixed breeds, two Beagles, two Basset Hounds, one Labrador Retriever; one Pincher; one Yorkshire Terrier; and one German Shepherd. Before randomization, two dogs left the study, of which one was Dachshund and one was a German Shepherd.

All dogs had spinal hyperesthesia at admission and were under the IES protocol. At T1 no dog had pain in both groups (COGI and BWSTT), which was assessed by palpation and recorded in slow motion mode.

After randomization (T1), dogs were divided into two groups (*n* = 10 each), with similar chondrodystrophy, age, and average weight.

For inter-observer validation, independent-samples Mann–Whitney *U*-tests were performed, revealing the absence of a statistical difference between observers in every moment of evaluation (*p* = 0.799).

In the total of 156 observations, that were performed and recorded between the two observers, there were only 21 inconsistencies, resulting in 13% inter-observer disagreement.

For comparison between groups and between evaluation time-points, a univariate analysis of variance was performed. The statistical model was considered significant (*p* < 0.001), with statistically significant differences between all OFS evaluation time-points (*p* < 0.001) and between groups (COGI × BWSTT) (*p* < 0.001). Both variables (group and time-point) also demonstrated a positive interaction (*p* = 0.019).

A higher mean OFS was observed in the BWSTT group (10.132) than in the COGI group (8.001) (Table 2).

**Table 2:** OFS total mean between BWSTT and COGI groups.

**Group**	**OFS mean**	**Std. error**	**95% confidence interval**	
			**Lower bound**	**Upper bound**
BWSTT	10.132	0.249	9.640	10.623
COGI	8.001	0.247	7.512	8.490

*OFS, Open Field Score; BWSTT, Body weight-supported treadmill training; COGI, Conventional overground intervention*.

In particular in T2 and T3, at each evaluation point there was a higher mean of OFS in the BWSTT group (Table 3). Additionally, it can be observed that, at 4 weeks after admission (T3), FNR-BWSTT dogs showed a borderline functional outcome (OFS ≥ 11), which was only demonstrated in the COGI group at T6 (after 7 weeks).

**Table 3:** T2 and T3 OFS evaluation in both groups.

**OFS moment**		**Mean**	**Std. error**	**95% confidence interval**	
				**Lower bound**	**Upper bound**
T2	BWSTT	8.300	0.589	7.136	9.464
	COGI	5.500	0.589	4.336	6.664
T3	BWSTT	10.700	0.589	9.536	11.864
	COGI	6.300	0.589	5.136	7.464

*OFS, Open Field Score; BWSTT, Body weight-supported treadmill training; COGI, Conventional overground intervention*.

**Figure 11:**
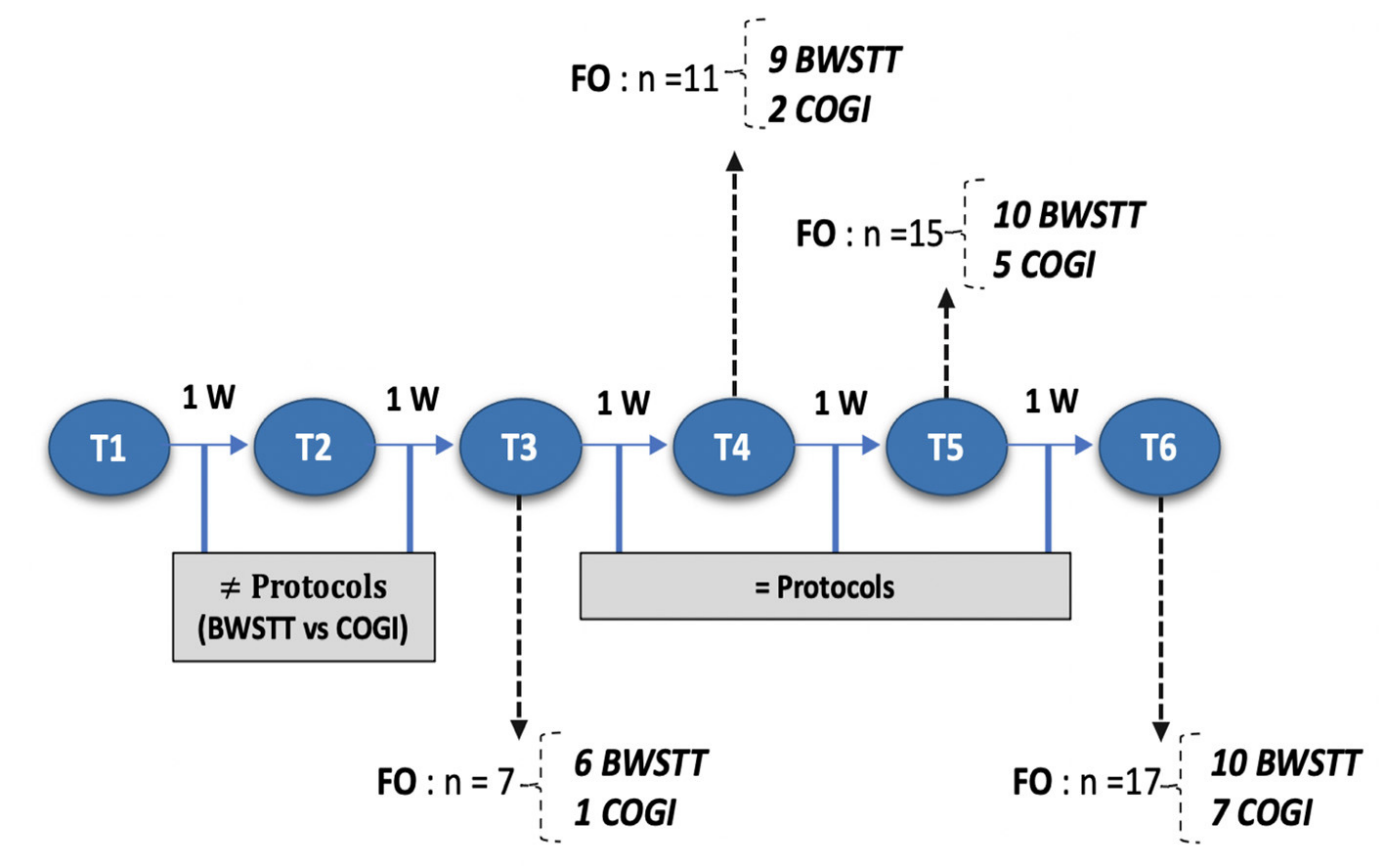
Flow diagram describing each moment according to number of weeks and functional outcome recovery. FNR, Functional neurorehabilitation; UWTM, Underwater treadmill; T1, After 2 weeks, randomization time; T2, After 1 week of specify protocol; T3, After 2 weeks of specific protocol; T4, T5, T6, After 3,4, and 5 weeks of the same protocol; BWSTT,Body-weight supported treadmill training; COGI, conventional overground intervation; FO, Functional outcome.

At T3, the functional outcome (OFS ≥ 11) was achieved within the BWSTT group in six dogs and in the COGI group in only one dog. The functional outcomes at the subsequent time-points (T4, T5, and T6) are described in Figure 11. In addition, at T3, the best result was seen in the BWSTT group, with four dogs classified as OFS 13, while one dog in the COGI group achieved only OFS 11.

Before T4 evaluation, one dog left the study by the owners' decision, thus the number of dogs decreased (*n* = 19), as shown in the flow diagram (Figure 10).

**Figure 12:**
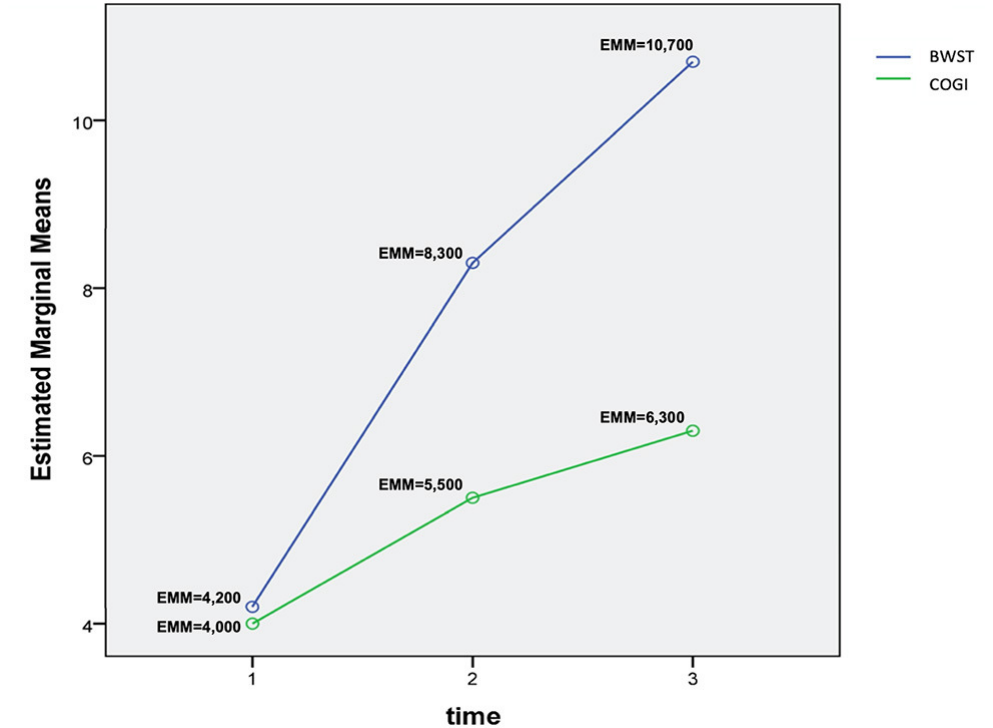
Interaction plot describing the evolution of OFS means in T1, T2, and T3 between groups. OFS, Open Field Score; BWSTT, Body weight-supported treadmill training; COGI, Conventional overground intervention; EMM, Estimated marginal means.

A two-way repeated measures ANOVA that compared the OFS mean differences between groups from T1 to T3 was done and the interaction plot obtained is shown in Figure 12. There was an interaction effect between time and treatment, thus OFS my vary based on time differently between the two treatments. Thus, there was a clear difference between the estimated mean of OFS after T1, with the highest difference observed at T3. According to the multivariate tests there was clear statistical significance between groups regarding treatment (*p* = 0.006) and during each evaluation time point (*p* = 0.000).

Upon medical discharge at the 7th week, a total functionality of 90% was achieved (17/19). Within the groups, functionality was 78% (7/9) in the COGI group by the 7th week, and 100 % (10/10) in the BWSTT group but already demonstrating these results in the 6th week (Figure 11).

Thus, for the COGI group, functional outcomes were achieved within a mean time of 6.1 weeks (range, 4–8 weeks) and for the BWSTT group within a mean time of 4.6 weeks (range, 3–6 weeks).

## Discussion

Our study population characterization agrees with that of most documented studies on IVDH Hansen type I, which report that the Dachshund is the breed most frequently diagnosed with this disease (59–63), followed by the French bulldog (64, 65). The main reason for this was explained by Hansen (66, 67) as involving transformation of the gelatinous nucleus pulposus into hyaline cartilage, which can begin as early as 2 months of age in Dachshunds and French Bulldogs.

In addition, chondrodystrophic breeds were the majority with 64% (14/22), in agreement with Kinzel et al. (68), and Aikawa et al. (5). However, 36.4% (8/22) were non-chondrodystrophic dogs, such as the German Shepherd and Labrador Retrievers, as described by Jeffery et al. (11) and Dewey and da Costa (36). The most frequent localization was the thoracolumbar region (69).

The average age of the affected dogs was 5.3 years, similar to that reported in previous studies (8, 62, 70), and the average weight was 9.65 kg, which was similar to that reported in earlier studies (65, 71, 72).

Chondroid metaplasia was an inclusion criterion. According to Ball et al. (73) and Thomas et al. (74), this should have demonstrated skewing of the sample population to males, but in our study, 55% (12/22) were females.

In T0, the 22 dogs started the FNR-UWTM protocol based on the IES technique to manage spinal hyperesthesia and locomotor training during 2 weeks. Spinal hyperesthesia manifestation decreased during this time (T0-T1), in particular in the 2nd week, and IES application was performed according to the pain level assessed by a correct technique of vertebral column palpation.

The repetitive locomotor training allowed stimulation of the muscle spindle and the cutaneous receptors during the bicycle movements performed by the technician, under water, in direct contact with the treadmill surface (22).

The treadmill locomotor training in incomplete SCI patients can induce plasticity in reflex pathways (75–77), as proven by Gossard et al. in incomplete SCI cats, who obtained functional plasticity of the locomotor CPGs (16).

The main goal of the FNR-UWTM training was to acquire the flexion–extension pattern before randomization (T1), equivalent to a minimum classification of OFS 3. In our study, this was not achieved in all dogs. Two dogs left the study before achieving this pattern (one with OFS 1; and one with OFS 2) while the remainder were randomly divided into the BWSTT and COGI groups (Figure 1).

At T1, the population was assigned to two groups (FNR-BWSTT × FNR-COGI) and dogs were evaluated, by two blinded observers using the OFS, resulting in a total of 156 observations with only 13% interobserver disagreement, confirming the reliability of the results obtained in the evaluations.

By a univariate analysis of variance, it was observed that after T1, both the BWSTT group and the COGI group showed a positive evolution over time in terms of an increase in OFS mean values (*p* < 0.001). Also, a two-way repeated measures ANOVA was performed to carefully evaluate from T1 to T3 and a similar significance was obtained (*p* = 0.007).

It is the authors opinion that just like in human medicine, repetitive step training for several consecutive weeks (by BWSTT or underwater treadmill) promotes an increase in muscle force output and endurance as well as an increase in rhythmic activity by the spinal locomotor circuitry and stimulation of remnant descending/ascending pathways (15). This selects the activation of motor neuron populations to obtain muscle synergy by descending motor pathway control and afferent input modulation, proposing rehabilitation strategies as a toll to improve stepping recovery after SCI (78).

Therefore, the limb displacement compensation mechanism, which involves increasing the hip angle, can cause activation of adductor muscles, and decreasing the angle can promote activation of the abductor muscles (79). In addition, the limb load compensation mechanism, which can stimulate stretch receptors and the Golgi tendon organs of extensor muscles, allows for faster rehabilitation in animal models (40, 80) and in humans (81, 82).

The interaction plot documented the biggest difference in the estimated means of OFS between groups in T3 (Figure 11), with an average of 10.7 in the BWSTT and 6.3 in the COGI groups (Table 2). In addition, the first time-point for assessing the difference between groups was at T2 (at 1 week after initiating differentiated training) with a clear distinction between them, this difference was maintained until T5, after 2 weeks of the same BWSTT training was implemented for ethical reasons.

At T5, the estimated OFS confidence interval showed an overlap of the upper limit of the COGI dogs and the lower limit of the BWSTT dogs, and functionality at this stage was considered to be 79% (15/19).

At the time of clinical discharge (T6), functional recovery was achieved in 90% (17/19) of all dogs and 100% had recovered bladder function. Within the BWSTT group, 100% (10/10) of the dogs recovered, while only 78% (7/9) in the COGI group recovered. The functional outcomes at the time of clinical discharge (90% functional recovery) agreed with the results of previous studies (1, 4, 8, 14, 73). However, in the BWSTT group, there was 100% functional recovery within an average time of 4.6 weeks. Considering that these dogs had severe grade I MFS, this protocol is a viable tool for implementation in sub-acute postsurgical IVDH dogs.

The results obtained in this population of subacute postsurgical IVDH dogs that had absent/decrease withdrawal reflex at admission, and a consequential descending pathway blockage compatible with spinal shock, showed that the underwater treadmill training could have promoted the appearance of the flexion/extension pattern. Also, the allocation of dogs in different groups demonstrated that in the BWSTT a functionality of 100% (10/10) was achieved within a mean time of 4.6 weeks, while in the COGI group 78% (7/9) were functional within 6.1 weeks, suggesting that even spontaneous recovery could occur in both groups, the BWSTT had the major success in recovery and in less time.

Recovery of function due to neurological injuries can be variable and difficult to predict. The most important challenge for the recovery of function is axonal regeneration and the formation of new functional synapses across the injury site (62, 83), mainly between the axons of damaged tracts and the new collateral axons (84). Thus, locomotor training is a strategy that promotes cutaneous reflex excitability (85). These can result from different mechanisms, involving the sprouting of undamaged fibers, regeneration of damaged fibers, and/or changes in synaptic efficiency (86, 87).

In humans BWSTT allows for greater trunk stabilization, a rhythmic timing exercise, and also assists with retropulsion of the stance phase, which promotes the hip extension critical to the initiation of the swing phase (88). Moreover, the combination of BWSTT and FES clearly improved walking speed, distance, limb coordination, strength, and muscle activation patterns (29). This combination showed significant improvements in cadence and step length and had a synergistic effect on muscle mass, balance, coordination, and rhythm. Complementary FES with low pulse frequency has been recommended for humans with incomplete SCI, probably because it facilitates training in standing (31, 46).

FES may also have been essential for its action in muscle atrophy and fatigue (89). Following SCI there are proportionally more fibers type II (fast-fatigable, anaerobic) than type I (slow, aerobic), which are also atrophied, leading to weakness and fatigue. This atrophy is also accompanied by a conversion of type I to type II muscle fibers. Thus, FES technique can increase contraction time and resistance to fatigue by reversing the muscle mass loss and the conversion of muscle fibers to a different type, probably resulting in more muscle fibers that become type I (90, 91). It also increases capillary density and blood flow, decreases spasticity and pain, increases the range of motion, and decreases contractures (92–95).

Research indicates that BWSTT promotes greater control of body posture and balance, both of which depend on organization of the multisensory system (96) and recruitment of muscles in the categories of the flexors/extensors and abductors/adductors (97). This may be among the main reasons for the faster recovery of dogs in the BWSTT group and also for the positive recovery of dogs who had been treated with the COGI protocol initially, but were switched to the BWSTT protocol. This last protocol leads to an increase in the amplitude and coordination of motor unit firing in leg muscles (23).

Our results indicated that COGI training could not promote as many repetitive exercises as BWSTT. For improved walking function, motor learning requires more than a simple repetition (20) and benefits from the opportunity to make and correct errors. This facilitates recovery of locomotion after incomplete SCI (17, 98).

Nevertheless, COGI dogs showed slow recovery, with positive evolution, probably due to the effects of walking training on the cutaneous muscular reflex, which comprises part of the flexor afferent pathway. This pathway is considered to be activated by excitatory and inhibitory interneurons within the CPGs (99).

In humans with incomplete SCI, it was reported no difference between BWSTT and COGI interventions. Likewise, no distinctions between the two approaches were seen from the kinematic and kinetic aspects (24, 28). This was not the case in our study, where BWSTT showed a markedly better performance. In human medicine, the combination of these types of training is considered to be beneficial (100). This is likely due to the ability of BWSTT to stimulate the stance phase and promote hip, knee, and ankle control, while COGI training allows integrated volitional control of all components of gait (101).

The biggest limitation of this study was the absence of a kinematic and kinetic evaluation, as well as electromyographic studies useful for neuromuscular monitoring, which could promote understanding of the differences between the groups. In addition, the decrease in the number of dogs that attended the first follow-up (*n* = 10) did not allow reliable assessment of the efficacy of these protocols in the long term. Finally, this study was also limited by the fact that dogs only differed in treatment for 2 weeks, as all dogs were transferred to the protocol with best results in T3 (BWSTT), which could be the cause of the overall differences between groups to be relatively small. Given these, further studies are warranted.

The difficulty in comparing differences between both groups is probably due to the fact that the dogs underwent different treatment for only 2 weeks.

## Conclusion

The differences in successful recovery between the BWSTT group and the COGI group were clearly demonstrated in this study. After 4 weeks of the FNR implementation 60% (6/10) had recovered in the BWSTT group, while 10% (1/10) had recovered in the COGI group.

Despite these differences, dogs in the COGI group managed to evolve positively, primarily after switching to the BWSTT protocol. This may suggest that conventional protocols can be applied to dogs with incomplete SCI, but can delay functional recovery, while treating dogs with SCI by BWSTT can yield better functional outcomes in less time.

Both protocols, which included FES and locomotor training, were considered to be safe and not harmful to any of the dogs, and promoted a positive evolution in dogs with grade I MFS incomplete SCI, diagnosed with T11–L3 IVDH Hansen type I, after surgery. Future studies should be continued with a focus on longer period times in a single treatment.

## Data Availability Statement

The raw data supporting the conclusions of this article will be made available by the authors, without undue reservation.

## Ethics Statement

The animal study was reviewed and approved by CEBEA—Faculty of Veterinary Medicine. Written informed consent was obtained from the owners for the participation of their animals in this study.

## Author Contributions

ÂM was the Certified Canine Rehabilitation Professional (CCRP) examiner/instructor at the University of Tennessee that performed all neurorehabilitation examinations and re-evaluations, and also the major contributor in writing the manuscript. DG and AC were members of the neurorehabilitation team that helped in the protocol execution and manuscript revision. AC was the second observer of this study. IV performed the statistical analysis. ÂM and AF designed the study protocol. AF revised all images recorded regarding the neurorehabilitation examinations with the help of ÓG. All authors have read and approved the final manuscript.

## Conflict of Interest

The authors declare that the research was conducted in the absence of any commercial or financial relationships that could be construed as a potential conflict of interest.
